# GPC-100, a novel CXCR4 antagonist, improves in vivo hematopoietic cell mobilization when combined with propranolol

**DOI:** 10.1371/journal.pone.0287863

**Published:** 2023-10-25

**Authors:** Devki D. Sukhtankar, Juan José Fung, Mi-na Kim, Thomas Cayton, Valerie Chiou, Niña G. Caculitan, Piotr Zalicki, Sujeong Kim, Yoonjung Jo, SoHui Kim, Jae Min Lee, Junhee Choi, SeongGyeong Mun, Ashley Chin, Yongdae Jang, Ji Yeong Lee, Gowoon Kim, Eun Hee Kim, Won-Ki Huh, Jae-Yeon Jeong, Dong-Seung Seen, Pina M. Cardarelli

**Affiliations:** 1 GPCR Therapeutics USA, Inc., Redwood City, California, United States of America; 2 GPCR Therapeutics Inc., Gwanak-gu, Seoul, Republic of Korea; 3 School of Biological Sciences, Seoul National University, Seoul, Republic of Korea; 4 Institute of Microbiology, Seoul National University, Seoul, Republic of Korea; Cincinnati Children’s Hospital Medical Center, UNITED STATES

## Abstract

Autologous Stem Cell Transplant (ASCT) is increasingly used to treat hematological malignancies. A key requisite for ASCT is mobilization of hematopoietic stem cells into peripheral blood, where they are collected by apheresis and stored for later transplantation. However, success is often hindered by poor mobilization due to factors including prior treatments. The combination of G-CSF and GPC-100, a small molecule antagonist of CXCR4, showed potential in a multiple myeloma clinical trial for sufficient and rapid collection of CD34^+^ stem cells, compared to the historical results from the standards of care, G-CSF alone or G-CSF with plerixafor, also a CXCR4 antagonist. In the present study, we show that GPC-100 has high affinity towards the chemokine receptor CXCR4, and it potently inhibits β-arrestin recruitment, calcium flux and cell migration mediated by its ligand CXCL12. Proximity Ligation Assay revealed that in native cell systems with endogenous receptor expression, CXCR4 co-localizes with the beta-2 adrenergic receptor (β_2_AR). Co-treatment with CXCL12 and the β_2_AR agonist epinephrine synergistically increases β-arrestin recruitment to CXCR4 and calcium flux. This increase is blocked by the co-treatment with GPC-100 and propranolol, a non-selective beta-adrenergic blocker, indicating a functional synergy. In mice, GPC-100 mobilized more white blood cells into peripheral blood compared to plerixafor. GPC-100 induced mobilization was further amplified by propranolol pretreatment and was comparable to mobilization by G-CSF. Addition of propranolol to the G-CSF and GPC-100 combination resulted in greater stem cell mobilization than the G-CSF and plerixafor combination. Together, our studies suggest that the combination of GPC-100 and propranolol is a novel strategy for stem cell mobilization and support the current clinical trial in multiple myeloma registered as NCT05561751 at www.clinicaltrials.gov.

## Introduction

Multiple myeloma (MM) is a leading hematological malignancy with an estimated 34,920 cases in the United States and approximately 588,161 cases worldwide each year [1]. Autologous Stem Cell Transplant (ASCT) is integral to the overall management of MM in eligible patients and has improved the anti-cancer response and survival compared to conventional chemotherapy [2–5]. The success of ASCT relies on harvesting a sufficient number of hematopoietic stem cells (HSC), which are predominantly obtained by mobilizing the HSCs from the bone marrow (BM) into the peripheral blood (PB) [6, 7]. In humans, HSCs are phenotypically characterized by the expression of CD34. A minimum of 2 x 10^6^ CD34^+^ cells/kg are essential for the HSC harvest, whereas the optimal number for improved engraftment and survival is >5–6 x 10^6^ CD34^+^ cells/kg [8, 9]. Granulocyte-colony stimulating factor (G-CSF) is a clinical standard of care for HSC mobilization [10]. However, G-CSF fails to mobilize optimal number of HSC in at least 40–50% MM patients [10, 11]. Some patients are treated with the combination of G-CSF and a small molecule CXCR4 antagonist plerixafor (AMD3100) [10]. Despite this combination treatment, 15–35% MM patients do not mobilize a sufficient number of cells [10, 12]. In a recent phase 3 clinical study, the combination of G-CSF and motixafortide, a peptide inhibitor of CXCR4, mobilized a significantly greater number of CD34^+^ cells compared to G-CSF plus placebo [13]. While this is promising, accumulating data suggests that MM therapies such as daratumumab [14] or lenalidomide [15] may negatively impact HSC mobilization. Mobilization failure can lead to potential loss of ASCT as a treatment option or significant toxicity from repeated mobilization attempts [16]. Moreover, G-CSF is contraindicated in conditions like sickle cell disease for stem cell collection [17] and exacerbates autoimmune diseases [18]. These factors warrant identification of novel strategies that not only address poor mobilization in MM patients, but also provide non-G-CSF options for adequate mobilization in MM as well as other disease indications [19, 20].

CXCR4 is a member of the chemokine G protein-coupled receptor (GPCR) family [21, 22] and is expressed on HSCs [21, 23]. CXCR4 signaling, mediated by its natural ligand CXCL12, plays a pivotal role in cellular chemotaxis, as well as retention and survival of HSCs in BM [23]. GPC-100, also known as Burixafor or TG-0054, is a novel small molecule antagonist with a high binding affinity to CXCR4. In a phase I trial with healthy volunteers, GPC-100 was well-tolerated and induced a 3- to 12-fold increase in circulating CD34^+^ HSCs compared to baseline following a single intravenous injection [24]. GPC-100, in combination with G-CSF, has been tested clinically in MM patients as an HSC mobilizer (NCT02104427) [25], and was shown to elicit a significant increase in HSCs with >5.0 x 10^6^ CD34^+^ cells/kg in 1–2 leukapheresis sessions [26]. This result was comparable with the historical results from G-CSF plus AMD3100 treatment.

Previous studies have demonstrated that CXCR4 physically interacts with the beta-2-adrenergic receptor or β_2_AR (gene ADRB2) in cell systems that overexpress both receptors [27–29]. Findings from Nakai et al suggest that β_2_AR selectively forms heteromeric complexes with CXCR4, and the stimulation of β_2_AR enhances CXCR4 signaling, potentially leading to increased lymphocyte retention in lymph nodes and decreased mobilization to PB [27]. β_2_AR is also expressed on HSCs [30, 31] and the adrenergic signaling plays a key role in regulating HSC niche in BM [32, 33]. Epinephrine and norepinephrine, the natural ligands of β_2_AR, were shown to influence the turnover and trafficking [34], as well as reduce the proliferative and differentiation capacity of the HSCs [35]. When human HSCs were co-stimulated with G-CSF and β_2_AR agonists, the expression of CXCR4 on HSCs increased, suggesting that the interactions between β_2_AR agonists and G-CSF in BM niche promote HSC retention by CXCR4 and impair mobilization by G-CSF [31].

Studies have noted the link between beta adrenergic inhibitor (beta blocker) usage and positive survival outcome in several cancer types including MM [36, 37]. The MM microenvironment is known to cause dysregulation of HSC function leading to changes in gene expression and altered hematopoietic differentiation [38, 39]. Effects of beta-adrenergic blockade on HSC differentiation in MM have been evaluated using propranolol, the FDA-approved non-selective beta blocker with a safe side effect profile [40]. A Phase II biomarker-driven randomized study showed that in MM patients undergoing ASCT, propranolol shifted cell differentiation away from the myeloid-lineage bias and toward the CD34^+^ HSC-like profile, which leads to enhanced engraftment [39]. In this study, activation of the sympathetic nervous system induced a shift in the basal gene expression profile towards a more inflammatory pattern termed as Conserved Transcriptional Response to Adversity (CTRA), which is associated with poor outcomes in ASCT [39]. Propranolol was shown to reduce the CTRA gene signature. In another study, BM samples from MM patients showed that propranolol can augment differentiation of HSCs into megakaryocyte-erythrocyte progenitors and reduce the number of granulocyte-monocyte progenitor cells, which are known to contribute to a pro-tumorigenic niche [41]. Together, these studies indicate that propranolol can block the negative effects of adrenergic signaling on HSC biology, induce HSC proliferation and differentiation, as well as synergize with a CXCR4 inhibitor considering the possible crosstalk between β_2_AR and CXCR4 in BM. Therefore, we speculated that the co-inhibition of CXCR4 and β_2_AR pathways can improve HSC mobilization.

In the present study, we report the in vitro characterization and in vivo mobilization efficacy of GPC-100 in comparison with AMD3100. We also provide evidence for the first time that in cancer cells endogenously expressing CXCR4 and β_2_AR, the two receptors co-localize and exhibit functional synergy. Furthermore, we demonstrate enhanced mobilization in vivo by GPC-100 in combination with propranolol and propose a new strategy for clinical application in stem cell mobilization.

## Methods

### Cell culture

MDA-MB-231, Namalwa and MM.1S cells were purchased from the American Type Culture Collection (ATCC) and U937 cells were purchased from the Korean Cell Line Bank (Seoul, Korea). Cells were cultured in RPMI 1640 with ATCC modification (Gibco, A1049101), supplemented with 10% fetal bovine serum (FBS) and 100 U/ml of Penicillin–Streptomycin.

### Construction of knock-out cells

Genscript provided non-targeting single guide RNA (sgControl: 5′-ACGGAGGCTAAGCGTCGCAA-3′), CXCR4-targeting sgRNA (sgCXCR4: 5′-ACTTACACTGATCCCCTCCA-3′), and ADRB2-targeting sgRNA (sgADRB2: 5’-CGTCTGCAGACGCTCGAACT-3’) that were cloned in pLentiCRISPRv2. Lentiviruses were produced and gene targeting was performed as described [42]. Namalwa-CXCR4 KO cells were used as a pool after puromycin selection. MDA-MB-231-ADRB2 KO clone was chosen using puromycin selection followed by limiting dilution. Flow cytometry was used to detect the surface expressions of CXCR4 and ADRB2 in the parental and KO cells with anti-CXCR4, anti-β_2_AR antibody or isotype control antibodies.

### Inhibition of CXCL12 binding to CXCR4

Competitive binding assays were performed using the TagLite^®^ system (CisBio). After CXCR4 terbium-labeled cells were plated, 5 μL of GPC-100 or AMD3100 were added, followed by 5 μL of 20 nM red fluorescently labeled CXCL12. After a 3-h incubation at room temperature, the plate was read on an EnVision plate reader (PerkinElmer). Inhibitory constants (Ki) for the respective compounds were determined by plotting the normalized HTRF ratio versus the compound concentrations using non-linear regression competitive binding “one site–fit Ki equation.” The HTRF ratio was calculated as the emission ratio of 665 nm/620 nm proportional to the amount of CXCL12 bound to CXCR4.

### Molecular docking of GPC-100 and AMD3100

The Schrödinger Small-Molecule Drug Discovery Suite was used for molecular modeling procedures, using the OPLS3e force field. The model of human CXCR4 in complex with small molecule antagonist IT1t (PDB: 3ODU) was imported and prepared using the Protein Preparation Wizard workflow. AMD3100 and GPC-100 were prepared using LigPrep on default settings. A total of 25 and 18 different conformations were generated for AMD3100 and GPC-100, respectively. The standard workflow for ligand docking in XP (extra precision) setting was followed for AMD3100 and GPC-100 to the CXCR4 structure [43]. Induced Fit Docking was also performed on GPC-100 [44]. To ensure proper docking of the binding pocket, the residues Y116/D171/Y255/E288 were selected as the center point. To avoid steric conflict with the large side chain Arg188, the trim option was selected. The top 20 poses for GPC-100 with an energy window of 30 kcal/mol were saved for further analysis.

### Calcium flux assay

MDA-MB-231 cells endogenously expressing CXCR4 and β_2_AR, or transduced with adenoviruses encoding CXCR4 for overexpression were used for calcium flux. Cells were cultured for 2 days prior to running the assay [45] and seeded at 8×10^4^ cells/well in a 96-well black clear-bottom microplate. The following day, cells were stained with Cal-520 AM (AAT Bioquest) for 2 h at 37°C and treated with antagonists for 30 min followed by agonist stimulation. Intracellular calcium flux was measured using a Flexstation 3 microplate reader (Molecular Devices).

### Migration assay

U937 or MM.1S cells were treated with GPC-100 or AMD3100 for 30 min and 100 μl of the cell suspension was added to a transwell insert with 8-μm pores. Cells were allowed to migrate toward CXCL12 in the presence or absence of the antagonists for 4 h (MM.1S) or 24 h (U937) at 37°C. The migration of U937 cells was measured using PrestoBlue Cell Viability Reagent (Invitrogen). For MM.1S cell line, the migrated cells were counted by flow cytometry. The fluorescence intensity (560 nm/590 nm excitation/emission) was detected using a Varioskan LUX Multimode Microplate Reader.

### PRESTO-Tango assay

CXCR4-Tango plasmid was gifted by Dr. Bryan Roth (Addgene plasmid # 66262) [46], and CXCR4-dV2-Tango plasmid was constructed by removing V2 tail of the V2 vasopressin receptor using Age I digestion. HTLA cells were kindly provided by Dr. Richard Axel (Columbia University, NY, USA) and cultured as described.[46] HTLA cells were seeded at 8×10^5^ cells/well in a 6-well plate. The following day, cells were transfected with CXCR4-dV2 Tango and pcDNA3.1-ADRB2 using Lipofectamine 3000 (Thermo Fisher Scientific). After a 48-h incubation, transfected cells were transferred to 96-well white bottom plates and incubated under serum-starvation for 4 h. Cells were pretreated with antagonist for 30 min, and then stimulated with agonist for 18 h. On day 5, cells were lysed with Steady-Glo solution (Promega) for 15 min at room temperature, and luminescence was measured with Varioskan LUX Multimode Microplate Reader.

### Proximity Ligation Assay (PLA)

MDA-MB-231 (parental and ADRB2 knockout) and Namalwa (parental and CXCR4 knockout) cells were fixed on microscope slides and treated with lambda protein phosphatase (New England Biolabs) for 1 h at 37°C. The NaveniFlex MR kit (Navinci) was used for blocking, primary antibody incubation, and PLA. Recombinant rabbit anti-CXCR4 antibody (clone UMB2, Abcam) and mouse anti-human β_2_AR antibody (clone E-3, Santa Cruz) were used as primary antibodies, while rabbit IgG and mouse IgG2b isotype (Abcam) were used as negative controls. Slides were mounted using VECTASHIELD Antifade Mounting Medium with DAPI and imaged using the IN Cell Analyzer 2500HS system (Molecular Devices).

### In vivo studies

#### Animals

Studies were performed at a facility accredited by the Association for Assessment and Accreditation of Laboratory Animal Care and in strict accordance with the recommendations in the Guide for the Care and Use of Laboratory Animals of the National Institute of Health. The protocol was approved by the Institutional Animal Care and Use Committee (IACUC) of Explora BioLabs (Protocol # EB17-010-141). C57BL/6J mice (female, 6–9 weeks old) were ordered from Jackson Laboratories and allowed to habituate for one week before handling. They were housed 5 per cage and had free access to food and water. Mice were randomized for each study so that all treatment groups contained similar age and weight distributions. Test articles were administered as shown in Table 1.

**Table 1:** Dosing for in vivo mobilization.

Treatment	Dose	Route	Volume	Dosing Frequency
GPC-100	30 mg/kg	Intravenous	5 mL/kg	Single injection
AMD3100	5 mg/kg	subcutaneous	10 mL/kg	Single injection
Propranolol	20 mg/kg	Intraperitoneal	10 mL/kg	Once a day (QD) x 7 days
G-CSF	0.1 mg/kg	subcutaneous	10 mL/kg	Twice a day (BID) x 5 days

GPC-100 and AMD3100 were administered alone or co-administered with propranolol on day 7. In the triple combination group, G-CSF was administered from days 2 to 6. CXCR4 antagonists and propranolol were injected 14 hours later. All compounds were reconstituted in PBS. Control mice received PBS in the same volume.

#### Sample collection

Mice were monitored daily and weighed prior to drug administration. Based on the body weight data, mice were randomized into groups using the randomization tool in Benchling Studies randomization software. PB was collected by submental bleeding (non-terminal) or cardiac puncture (terminal). For terminal blood collections, mice were placed in an induction chamber with 2.5–4% isoflurane and then a maintenance dose of 2–3% through a nose cone. A toe pinch on all four quadrants was applied to confirm the surgical plane of anesthesia. Upon completion of terminal blood collection from cardiac puncture, the mice were euthanized via cervical dislocation while still under the effects of isoflurane. Approximately 25 μL blood was used for CBC analysis and the remaining blood was processed for flow cytometry or colony forming unit (CFU) assay. For non-terminal submental bleeding, a 4mm lancet was used to puncture the linguofacialis vein under the chin. Blood was collected at 1 or 2 h post GPC-100 and AMD3100, and 14 h post G-CSF. For the time-course of mobilization, each mouse was bled twice. Submental bleeding was performed at 0.5 h, 1 h or 2 h after the respective dosing and cardiac puncture was performed at 3 h, 4 h and 6 h post-dose. All doses of the treatments used in the study were well tolerated and no clinical observations were noted during the procedures.

#### CBC analysis

Blood samples were processed for complete blood count (CBC) analysis using the Abaxis VetScan HM5 hematology analyzer (Abaxis), which reports 18-parameters including WBC, lymphocytes, neutrophils, monocytes, platelets, hemoglobin, RBC and morphology.

#### Flow cytometry

Mobilization of mouse HSC, characterized as LSK cells (Lineage- Sca-1+ c-Kit+) [47], was determined in two separate studies. For both studies, mononuclear cells were isolated from PB post-CBC analysis and stained with anti-lineage cocktail, c-Kit and Sca-1 antibodies for the first study (S3 Table in S1 File). For the second study, CD150 or CD34 antibodies were added to determine HSCs with long-term repopulating capacity (LT-HSC) (CD150^+^ or CD34^-^ LSK) [48]. Samples were acquired with a Cytek Aurora spectral flow cytometer (Fremont, CA) and data was analyzed with CellEngine software. Gating was determined using FMO (Fluorescence Minus One) controls. The percentage of c-Kit^+^ Sca-1^+^ cells as a subset of parent Lin- cells were used to determine the total number of LSK cells μL of blood. For the second study, frequency of CD150^+^ or CD34^-^ cells was measured as a subset of parent LSK cells. Number of LSK cells in peripheral blood were normalized based on the WBC count obtained in that study.

#### Colony Forming Unit (CFU) assay

8 x 10^5^ mononuclear cells isolated from PB post-CBC analysis were added to tubes of semisolid methylcellulose medium (StemCell Technologies) known to support erythroid and myeloid progenitors [49]. Seven days later, colonies showing appearance of granulocyte-monocyte progenitors (CFU-GM) and burst forming erythroid units (BFU-E) formed and were counted by a blinded experimenter. Total CFU were calculated as a total number of CFU-GM and BFU-U colonies.

#### Statistical analyses

Data analyses were performed using GraphPad Prism and all data are presented as mean ± SEM. Comparisons of data across two dosing conditions were made using the Mann-Whitney test or multiple dosing conditions using the one-way ANOVA with Turkey’s multiple comparison test. P < 0.05 was considered statistically significant for all tests. * ≤ 0.05, ** ≤ 0.01, *** ≤ 0.001, **** ≤ 0.0001.

List of all reagents and sources used in this study are listed in the supplementary section.

## Results

### GPC-100 shows distinct binding modes and higher binding affinity for CXCR4 compared to AMD3100

**Fig 1:**
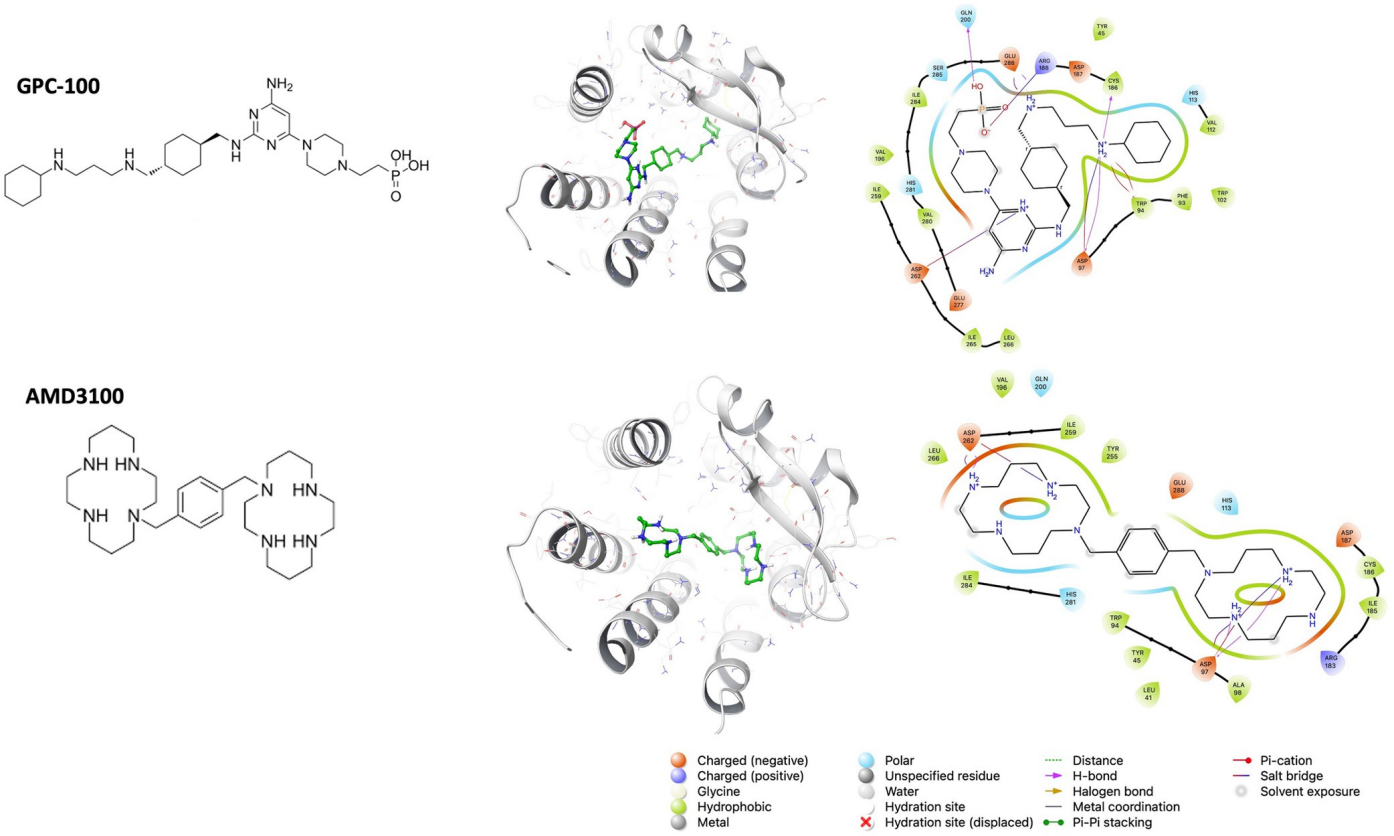
Comparison of CXCR4 binding between GPC-100 and AMD3100. (A) Chemical structures of AMD3100 and GPC-100. (B) Overlay of the proposed binding modes for GPC-100 and AMD3100 obtained from molecular docking with the template of an inactive structure of CXCR4 and induced-fit algorithm from Schrödinger. CXCR4 transmembrane helices are shown and annotated as gray ribbons. 2D ligand interaction diagram presents residues colored triangular picks. Picks that are pointing away represent backbone of residue facing towards the respective ligand. Picks facing towards the respective ligand represent side chain of residue facing the ligand.

The chemical composition of GPC-100 demonstrates distinct divergence from AMD3100 (Fig 1a). To understand these differences at a molecular level, we use in silico docking experiments of the two antagonists to an inactive structure of CXCR4. Previous studies with various CXCR4 antagonists have identified major and minor pockets of interactions within the CXCR4 orthosteric binding site [43]. The induced-fit algorithm from Schrödinger was used for GPC-100 [43, 44]. Previously validated residues with secondary amines, namely Asp97 and Asp262 [50], contributed to interactions of GPC-100 and AMD3100 with CXCR4. However, the phosphoryl group of GPC-100 that is absent in AMD3100 makes an ionic interaction with Arg188, and hydrogen bond with Gln200 (Fig 1b). These energetically favorable interactions suggest enhanced anchor points for GPC-100 that are absent in AMD3100.

**Fig 2:**
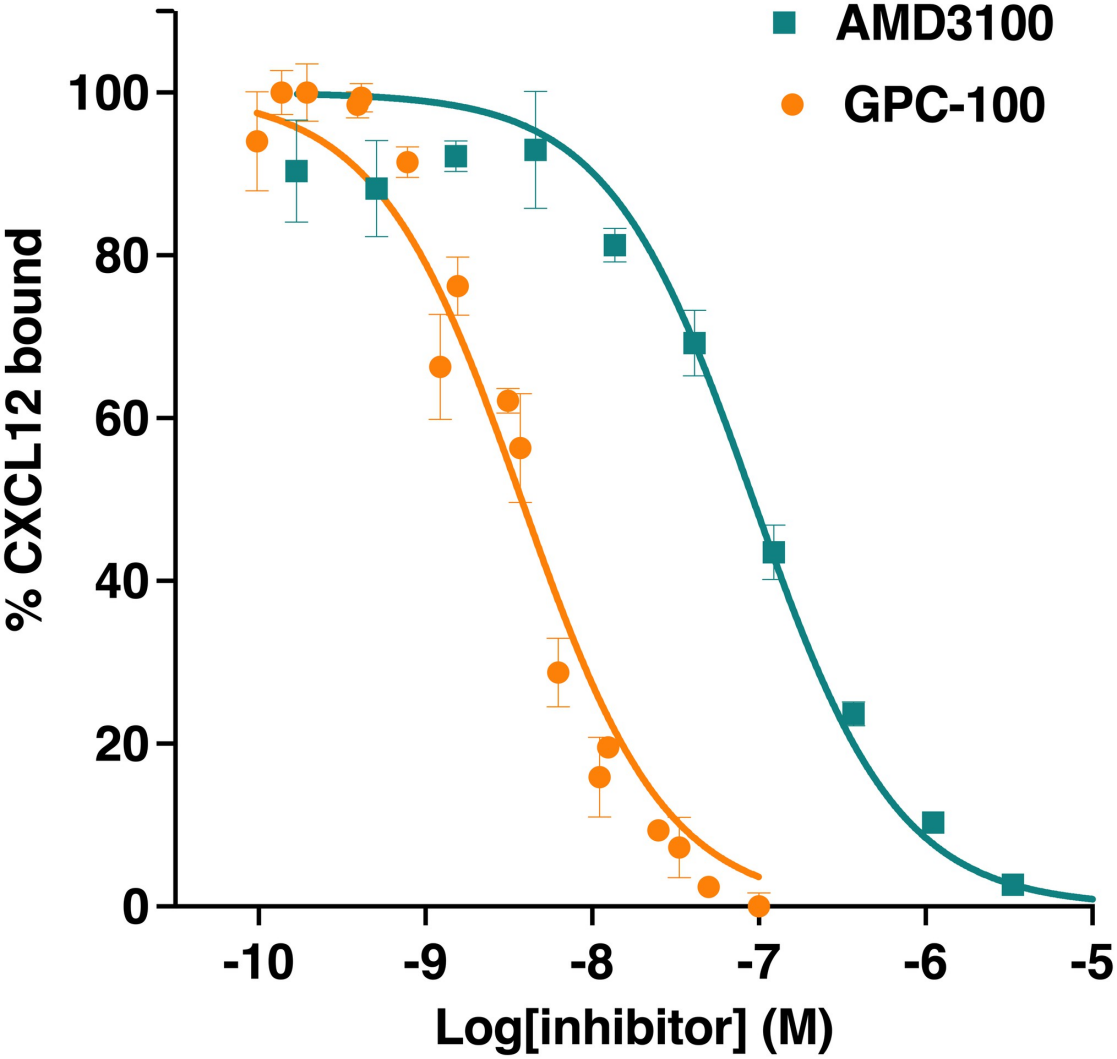
Dose response curves showing competitive inhibition of CXCL12 binding to CXCR4 by GPC-100 and AMD3100. Ki for the respective compounds were determined by plotting the normalized HTRF ratio (% CXCL12 bound) versus the compound concentrations and using non-linear regression competitive binding “one site–fit Ki equation.” Data expressed as mean ± SEM.

We tested how these structural differences translate to ligand binding ability using the TagLite^®^ technology. Dose response curves of both GPC-100 and AMD3100 demonstrated competitive inhibition of CXCL12 binding to CXCR4 (Fig 2) with the inhibitory constant (Ki) of GPC-100 as ~ 1.6 nM and AMD3100 as ~ 40 nM. This indicates that GPC-100 has a higher affinity for CXCR4 than AMD3100 (Fig 2).

### Pharmacological characterization of GPC-100 demonstrates its efficacy in inhibiting the CXCR4/CXCL12 axis

**Fig 3:**
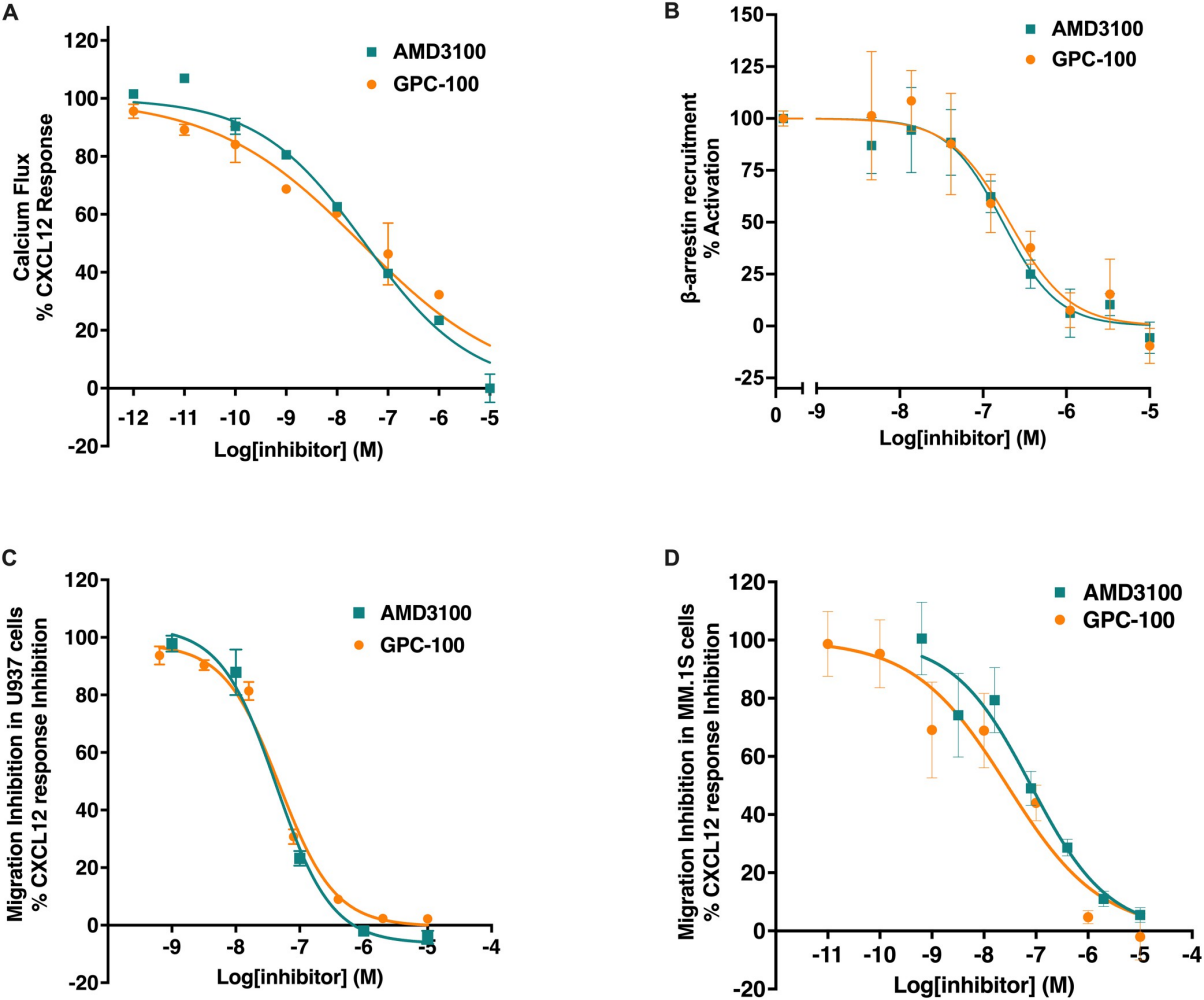
Pharmacological inhibition of CXCR4/CXCL12 axis by GPC-100 and AMD3100. (A) Inhibition of CXCL12 (20 nM)-induced calcium flux by GPC-100 and AMD3100 in MDA-MB-231 cells transduced with CXCR4. (B) Inhibition of CXCL12 induced β-arrestin recruitment to CXCR4 by GPC-100 and AMD3100 using Presto-Tango assay in HTLA cells overexpressing CXCR4-tango and β_2_AR. Inhibition of CXCL12 induced migration of (C) U937 and (D) MM.1S cells by GPC-100 and AMD3100. Data expressed as mean ± SEM.

We implemented several key assays to determine the effects of GPC-100 on CXCR4 signaling. First, we utilized calcium flux assay in MDA-MB-231 cells overexpressing CXCR4 (S1 Table in S1 File), which yielded a robust calcium flux response when stimulated with CXCL12. Dose response titration of GPC-100 and AMD3100 led to a dose dependent inhibition of CXCL12-induced calcium flux with IC_50_ values of 30 nM and 35 nM, respectively (Fig 3a). We also measured the recruitment of β-arrestin to CXCR4, which is known to occur upon CXCL12 binding [51–53]. In the PRESTO-TANGO β-arrestin assay [54], both GPC-100 and AMD3100 inhibited CXCL12 mediated recruitment of β-arrestin to CXCR4 with IC_50_ values of 207 nM and 172 nM, respectively (Fig 3b). Directly relevant to stem cell mobilization, cell migration is a key phenotypic response upon CXCR4 activation [55–57]. For this assay, we used MM.1S (multiple myeloma) and U937 (acute myeloid leukemia) cells with high endogenous CXCR4 expression (S1 Table in S1 File, S1 Fig in S2 File) [58], which showed a robust migration response to CXCL12 with a sub-nanomolar EC_50_ values (S2a, S2b Fig in S2 File). In U937 cells, both GPC-100 and AMD3100 efficiently blocked CXCL12-mediated migration with IC_50_ of 48 nM and 42 nM, respectively (Fig 3c). In MM.1S cells, CXCL12-induced migration was blocked by GPC-100 with a IC_50_ value of 28 nM, and AMD3100 with a IC_50_ value of 80 nM (Fig 3d).

### Endogenously expressed CXCR4 and β_2_AR reside in close proximity in a model cell system

**Fig 4:**
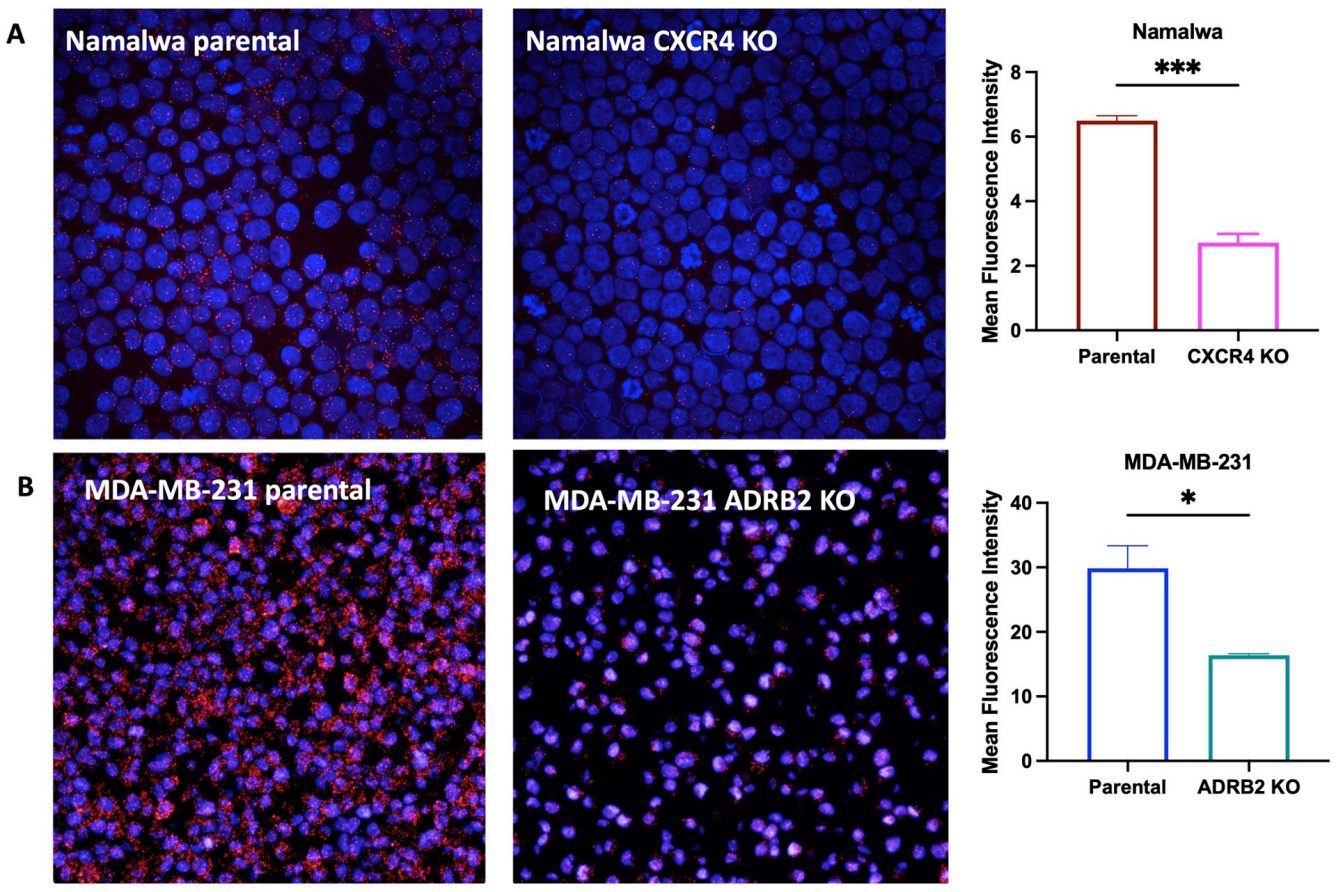
CXCR4 and β_2_AR co-localization in cancer cells. PLA showing proximity between CXCR4 and β_2_AR in (A) Namalwa parental and Namalwa CXCR4 knock-out cells, (B) MDA-MB-231 parental and MDA-MB-231 ADRB2 knock-out cells. All images are presented at 40X magnification. Data expressed as mean ± SEM with statistical significance for MFI (mean fluorescence intensity).

Previous studies in cardiomyocytes and lymphocytes suggest that CXCR4 and β_2_AR form heteromers [27]. Here, we investigated CXCR4 and β_2_AR heteromerization in a native state, wherein the receptors are endogenously expressed. To test this, we applied PLA, which allows for the detection of protein-protein interactions using endogenous and non-engineered receptors [59]. Because the PLA detects the amplified DNA as dots, only a few interacting molecules can produce a robust signal, making it a sensitive and specific assay. We used Namalwa and MDA-MB-231 cells as model cancer cell lines chosen based on receptor mRNA transcript levels, surface expression and cell-type diversity (S1 Table in S1 File, S3 & S4 Figs in S2 File). Using primary antibodies specific to the intracellular domains of CXCR4 and β_2_AR, we showed a positive PLA signal (over 5 PLA events per cell) in Namalwa cells, indicating stable proximity of endogenous CXCR4 and β_2_AR receptors (Fig 4a). To confirm specificity of receptor proximity, we constructed a knockout of CXCR4 in Namalwa and confirmed significant decrease in surface CXCR4 expression (S3a & S4b Figs in S2 File). PLA signal was significantly decreased in CXCR4 knockout Namalwa cells compared to the parental cells (Fig 4a). While these results were encouraging, the relatively low abundance of β_2_AR in Namalwa made detection by PLA challenging. We next investigated MDA-MB-231 cells that express higher levels β_2_AR mRNA (S1 Table in S1 File). In this cell system, the PLA yielded a strong signal of about 30 events per cell (Fig 4b). The specificity of the PLA was confirmed in a β_2_AR knock-out (S4c & S4d Fig in S2 File) of MDA-MB-231 cells (Fig 4b) suggesting that parental CXCR4 and β_2_AR exist in close proximity in a native cell environment.

### Synergistic pharmacology upon co-activation of CXCR4 and β_2_AR that is inhibited by GPC-100 and propranolol

**Fig 5:**
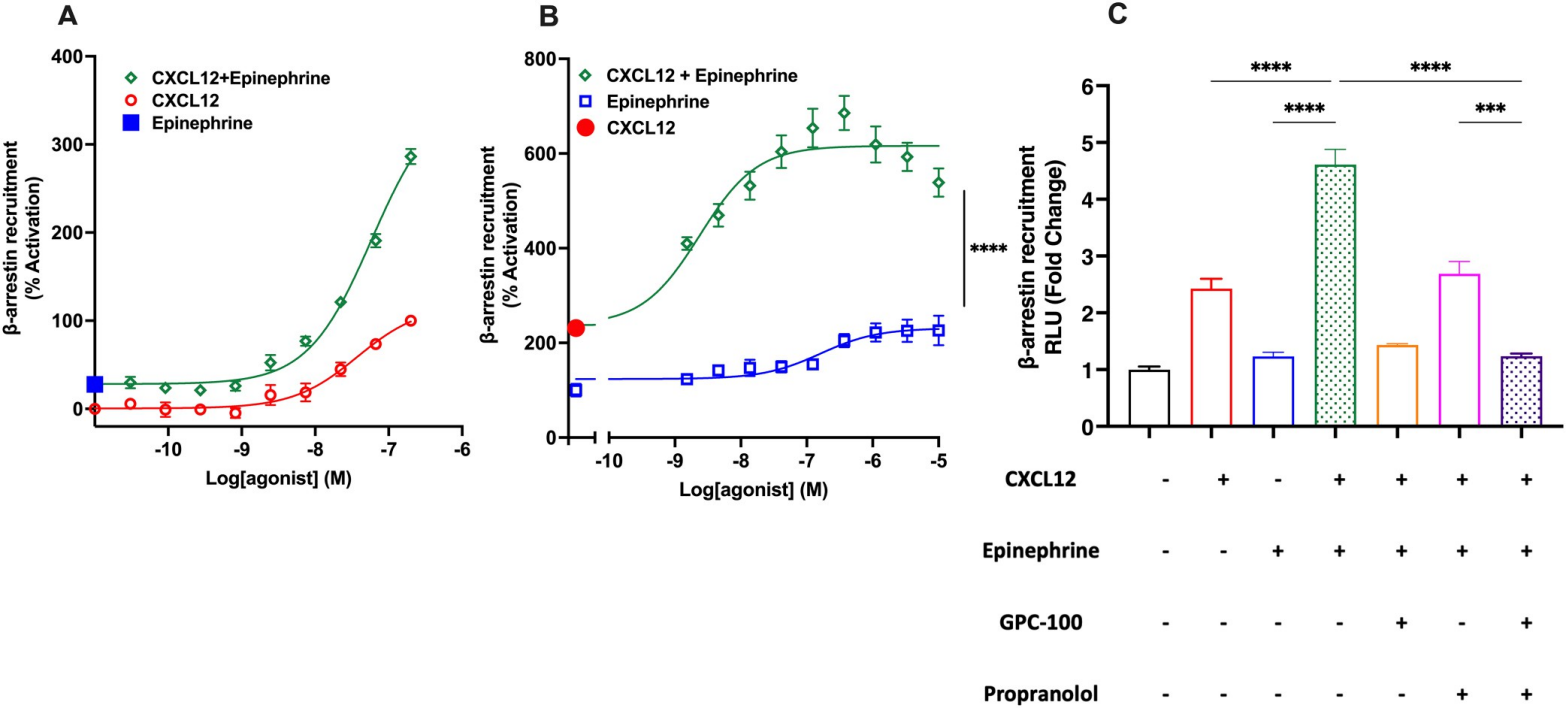
Synergistic increase in β-arrestin recruitment to CXCR4 by CXCR4 and β2AR co-activation. HTLA cells overexpressing CXCR4-tango and β_2_AR were treated with (A) 0–200 nM CXCL12 and 1 μM epinephrine or (B) 100 nM CXCL12 and 0–10 μM epinephrine in two separate experiments. Co-treatment with epinephrine and CXCL12 led to synergistic increase in β-arrestin recruitment. (C) Fold-change in β-arrestin recruitment to CXCR4 by 100 nM CXCL12, 400 nM epinephrine, agonist co-treatment, 10 μM GPC-100, 10 μM propranolol or antagonist co-treatment. Data expressed as mean ± SEM.

Functional effects of CXCR4 and β_2_AR proximity were evaluated in downstream signaling assays using receptor co-activation and co-inhibition. β-arrestins act as intracellular scaffolds that mediate receptor desensitization, internalization, and G-protein independent signaling [60–62]. In HTLA cells overexpressing CXCR4-tango and β_2_AR, CXCL12 induced β-arrestin recruitment to CXCR4 as expected (Fig 5a). Epinephrine also induced a modest but meaningful β-arrestin recruitment to CXCR4 (Fig 5b). However, co-treatment of these cells with CXCL12 and epinephrine led to a significant synergistic increase of β-arrestin recruitment compared to CXCL12-only treatment (Fig 5c, E_max_ 286 ± 12.20% with co-treatment), which was blocked by GPC-100 and propranolol combination (Fig 5c).

**Fig 6:**
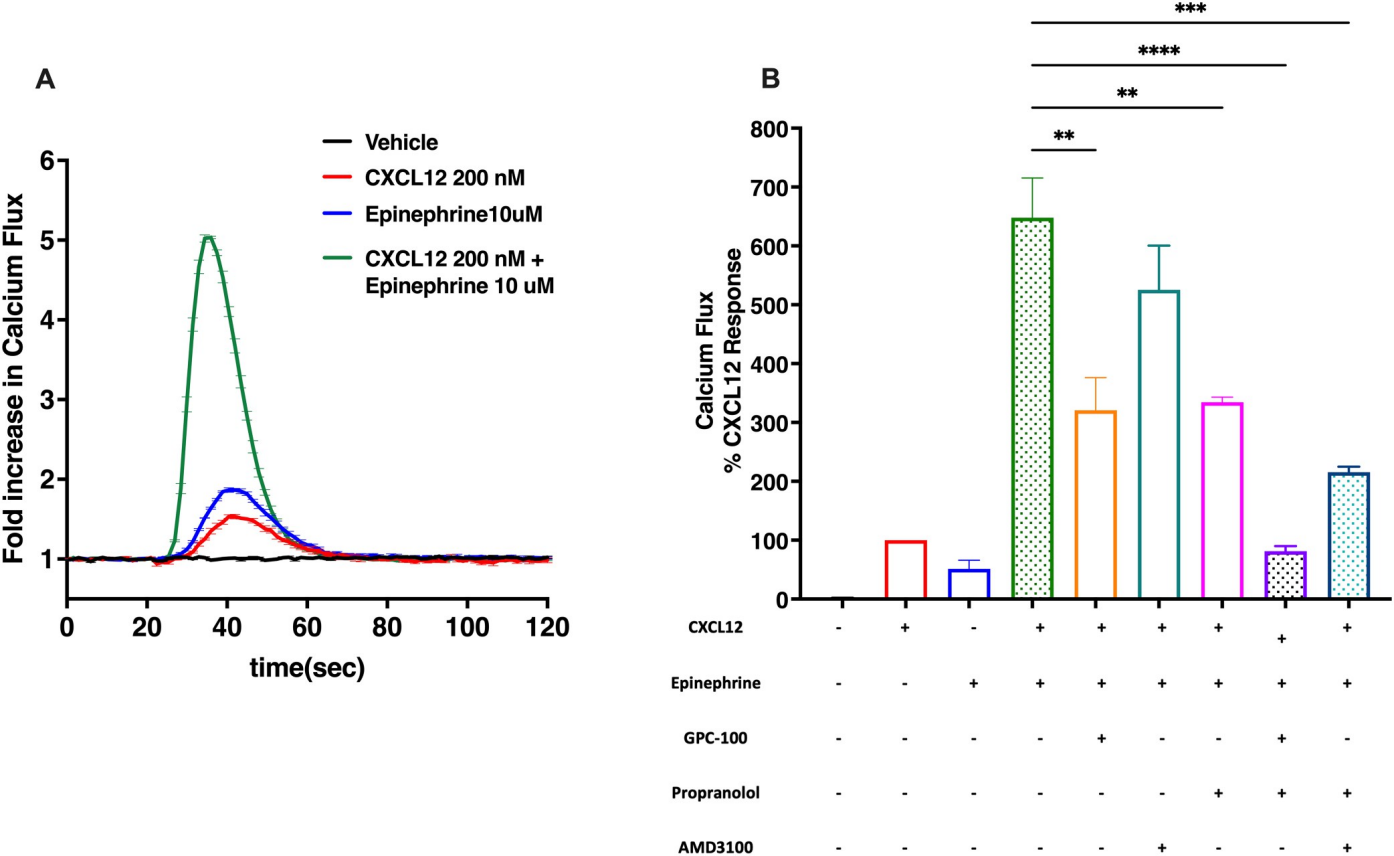
Effect of co-treatment with GPC-100 and propranolol on CXCL12 and epinephrine-induced crosstalk. (A) Calcium flux in MDA-MB-231 cells endogenously expressing CXCR4 and β_2_AR by vehicle, 200 nM CXCL12, 10 μM epinephrine or their co-treatment. Data indicate fold-increase compared to CXCL12. (B) Inhibition of synergistic calcium increase by 10 μM GPC-100, 10 μM AMD3100, 10 μM propranolol or their co-treatment in MDA-MB-231 cells treated with CXCL12 and epinephrine. Percent calcium flux was normalized to CXCL12. Data expressed as mean ± SEM with statistical significance.

Treatment of MDA-MB-231 cells endogenously expressing CXCR4 and β_2_AR (S5 Fig in S2 File) with CXCL12 or epinephrine alone elicited a modest calcium flux response. However, co-treatment with CXCL12 and epinephrine resulted in a synergistic increase in the calcium flux (Fig 6a). This was partially inhibited by the single antagonist treatment with GPC-100, AMD3100 or propranolol (Fig 6b). However, complete blockade of calcium flux was only achieved with GPC-100 and propranolol co-treatment. On the other hand, AMD3100 and propranolol combination resulted in partial inhibition of synergistically increased calcium flux. Therefore, GPC-100 was a more potent CXCR4 antagonist when combined with propranolol in this assay. These findings also suggest that CXCR4 and β_2_AR existing in close proximity can be co-activated to functionally regulate the downstream calcium signaling pathway, as well as affect the receptor internalization machinery, and potentially, the scaffolds that mediate the G-protein independent non-canonical signaling pathways.

### Combination treatments with GPC-100 and propranolol improve mobilization in mice

**Fig 7:**
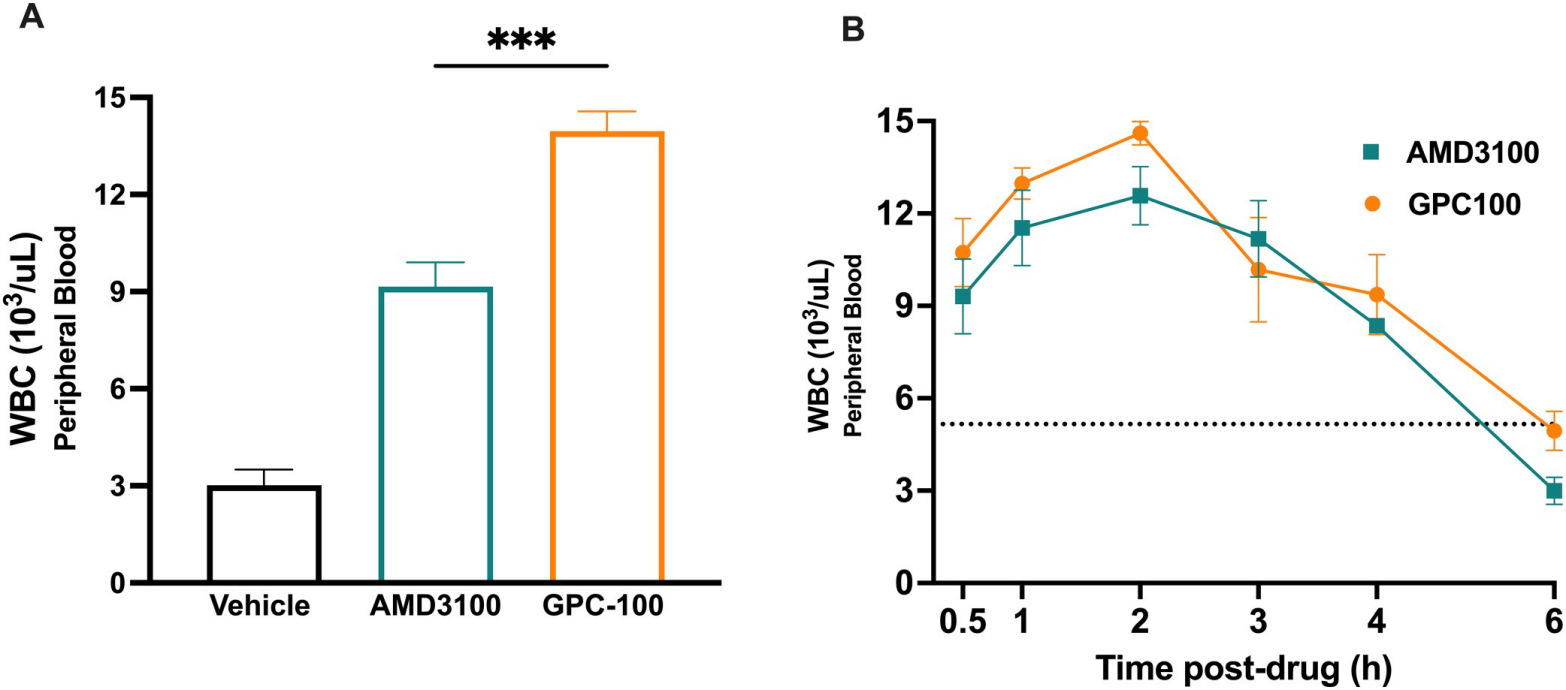
In vivo mobilization by GPC-100 or AMD3100. (A) WBC mobilization to PB followed by single injection of GPC-100 (30 mg/kg, IV) or AMD3100 (5 mg/kg, SC). PB was collected 1-hour post-drug. (B) Time course of mobilization by GPC-100 or AMD3100. PB was collected at various time points post-drug. Each mouse was bled twice. The bleeding at 0.5h, 1h and 2h was non-terminal, whereas at 3h, 4h, 5h it was terminal. A and B refer to two independent studies. Data expressed as mean ± SEM.

We determined GPC-100 induced mobilization compared to AMD3100 at the most efficacious doses for both antagonists. Numerous studies in mice report the peak mobilization by AMD3100 (5 mg/kg, SC) at 1 hour [63–65]. Therefore, PB WBC counts were determined at 1 hour following GPC-100 and AMD3100 administration. GPC-100 produced a 4.6-fold increase, whereas AMD3100 produced a 3-fold increase in circulating WBCs compared to the vehicle (Fig 7a). We then compared the time-course of mobilization between GPC-100 and AMD3100 from 30 min- to 6 hours post-drug. While both antagonists showed peak mobilization at 2 hours, GPC-100-induced mobilization was greater than AMD3100 (Fig 7b). Based on this time-course, PB was collected at 2 hours post-GPC-100 in the following studies.

**Fig 8:**
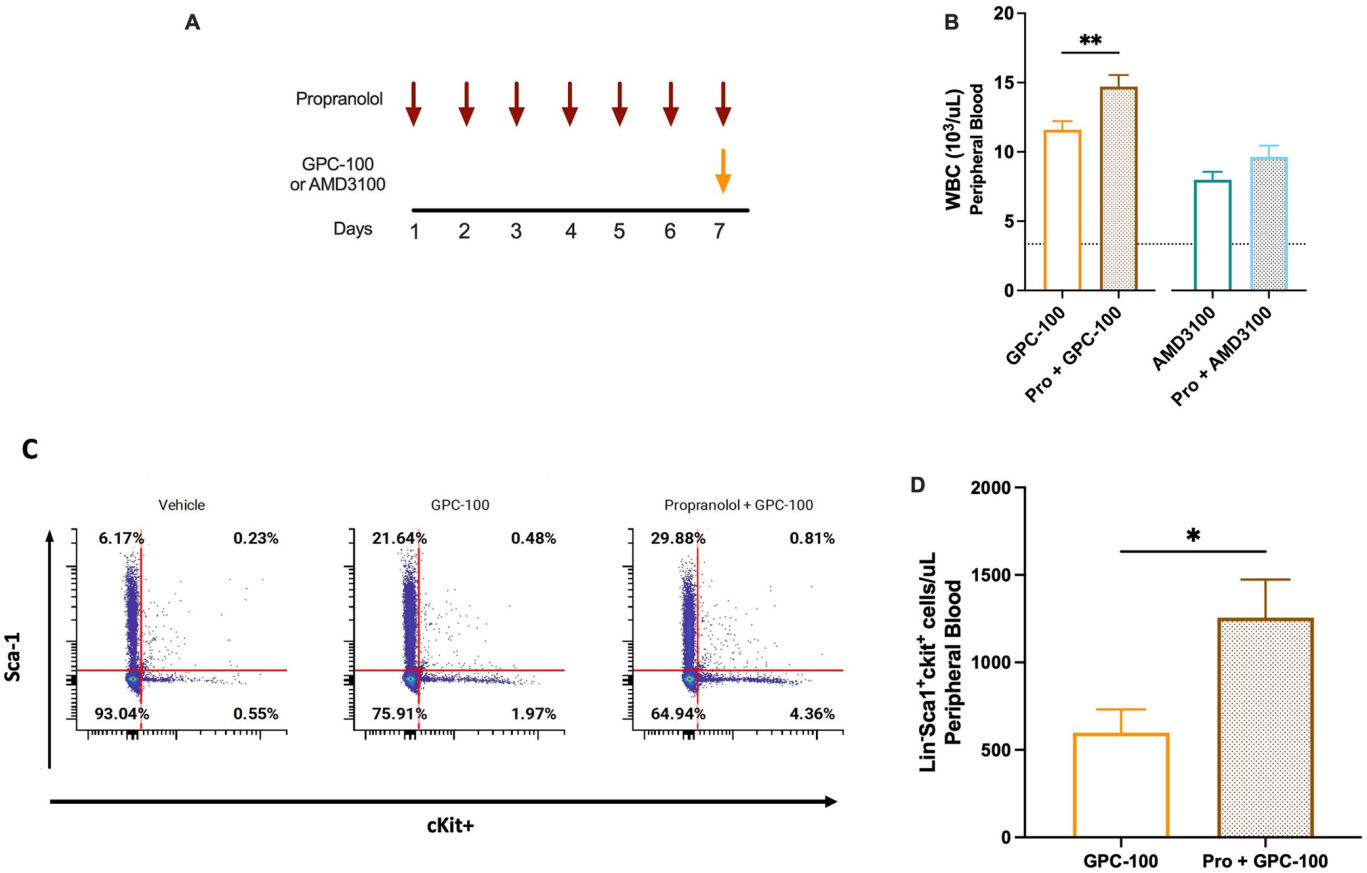
In vivo mobilization by GPC-100 and propranolol. (A) Dosing regimen indicating propranolol (20 mg/kg, IP) pretreatment for 7 days, followed by GPC-100 (30 mg/kg, IV) co-administration on day 7. AMD3100 (5 mg/kg SC) was similarly co-administered on day 7 with propranolol. (B) WBC mobilization by GPC-100 and propranolol, and AMD3100 and propranolol combinations. Dotted line indicates average WBC counts in vehicle-treated mice (C) Representative LSK frequency analysis in PB (D) number of mobilized LSK cells in mice treated with PBS, GPC-100 alone or in combination with propranolol. Data from two separate experiments. Pro: Propranolol. Data expressed as mean ± SEM.

To evaluate the impact of β_2_AR blockade in vivo, mice were administered propranolol. Propranolol dose was selected based on the dose titration (5–40 mg/kg, IP) when combined with GPC-100 (S6 Fig in S2 File). Pretreatment with propranolol (20 mg/kg, IP) over 7 days (Fig 8a) improved GPC-100 induced mobilization by 27% (Fig 8b). Propranolol also enhanced AMD3100-induced mobilization (Fig 8b). In particular, propranolol improved lymphocyte mobilization for both antagonists (S7a & S7c Fig in S2 File). Further experiments to determine HSC mobilization revealed that 7-day propranolol pretreatment enhanced LSK cell mobilization by GPC-100 resulting in a 4-fold increase in circulating HSCs (Fig 8c and 8d).

**Fig 9:**
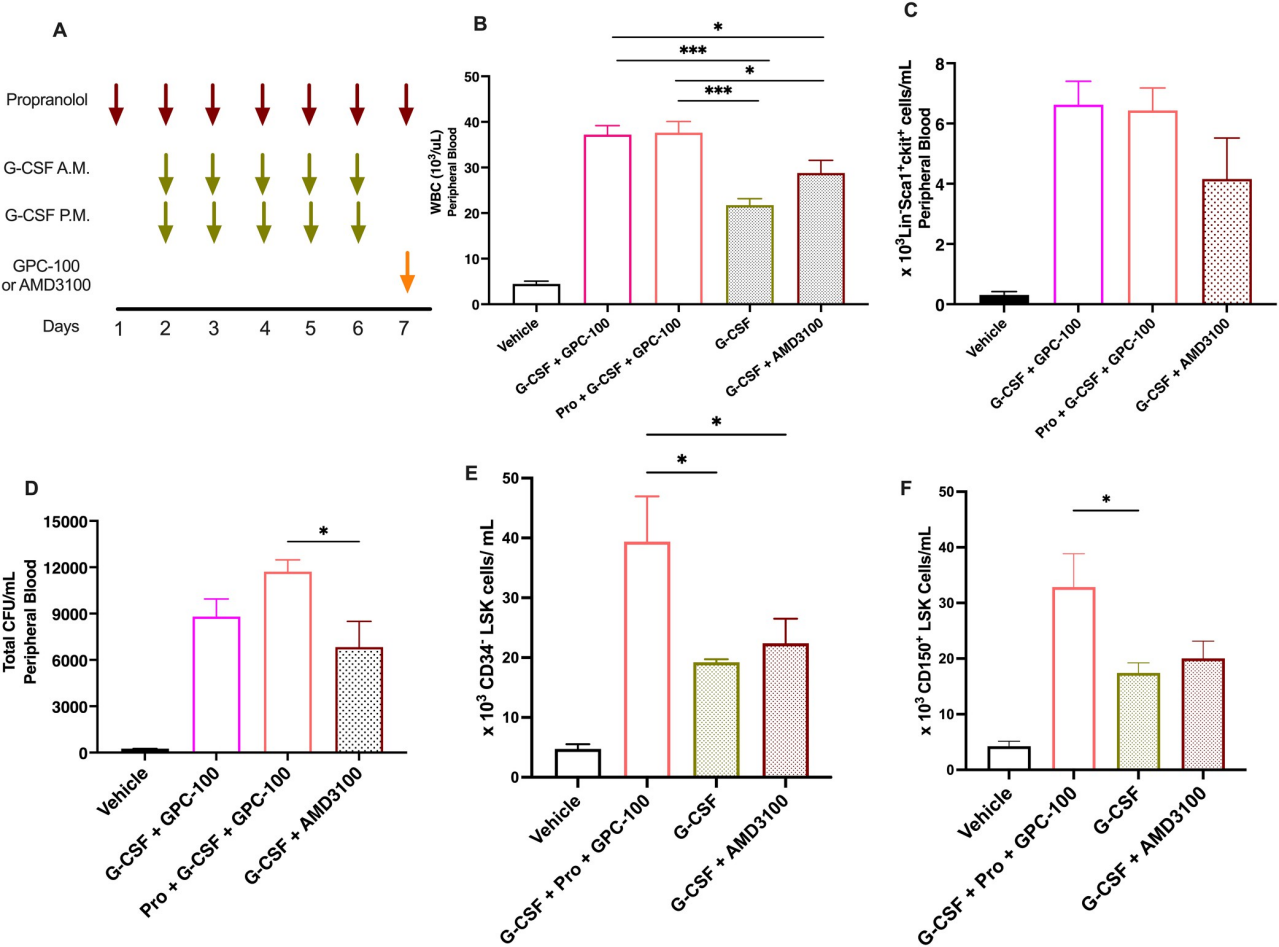
In vivo mobilization by the triple combination of GPC-100, propranolol, and G-CSF. (A) Dosing regimen. G-CSF (0.1 mg/kg, SC) was administered 5 days two-times a day (BID) from day 2 to day 6, propranolol (20 mg/kg, IP) was administered once daily for 7 days. GPC-100 (30 mg/kg, IV) was co-administered with propranolol on day 7. Mice in the triple combination group received all three treatments. For comparison with standards of care, G-CSF was administered alone or with AMD3100 (5 mg/kg SC) administered on day 7, 12 h post G-CSF. (B) WBC mobilization, (C) mobilization of LSK cells (Lin-Sca1+ckit+) evaluated by flow cytometry, (D) Number of total CFU in PB, (E) mobilization of CD34^-^ LSK cells and (F) CD150^+^ LSK Cells. Flow cytometry analyses and the CFU assay were performed on different cohorts of age- and weight-matched mice. Each mouse is assayed individually. Pro: Propranolol, G: G-CSF. Data expressed as mean ± SEM.

In the following studies, mobilization by the triple combination of G-CSF, GPC-100 and propranolol was compared with the current ASCT standards of care, i.e., G-CSF alone or in combination with AMD3100. The triple combination (Fig 9a) as well as the combination of G-CSF and GPC-100 induced an 8.2- and 8.4-fold increase in WBC mobilization, respectively, that was significantly greater compared to the increased WBC count by G-CSF alone (4.5-fold) or G-CSF plus AMD3100 (6.6-fold) (Fig 9b). Analysis of HSC or LSK cell mobilization revealed that G-CSF plus AMD3100 treatment resulted in a 13-fold increase in LSK cells in PB compared to the vehicle. In comparison, G-CSF and GPC-100 combination with and without propranolol resulted in 20-fold and 24-fold increase in LSK cells, respectively (Fig 9c).

We then compared the functional capacity of cells mobilized by the triple combination in a CFU assay. CFU assay was conducted to measure the mobilized HSCs based on their ability to form CFU-GM and BFU-E colonies. The triple combination produced a 47-fold increase in total CFUs over vehicle control compared to a 35-fold and 27-fold increase over vehicle from G-CSF plus GPC-100 and G-CSF plus AMD3100 treatments, respectively (Fig 9d). Addition of propranolol to G-CSF and GPC-100 combination increased mobilization of the LT-HSCs characterized as CD34-CD150+CD48+LSK (S8 Fig in S2 File). Therefore, we compared mobilization of LT-HCS by triple combination with the standards of care by analyzing CD150+ LSK cell population as well as primitive CD34-LSK population [48, 66]. The triple combination mobilized significantly more LSK cells that lack CD34 or express CD150 (Fig 9e and 9f). This indicates that the addition of propranolol can improve mobilization of primitive and functionally capable HSCs. The pattern of LSK and CFU counts across the different drug combinations was consistent with the WBC count from matched samples, supporting the use of WBC counts as a surrogate marker for stem cell mobilization.

## Discussion

In the present study, we provide in vitro and in vivo characterization of GPC-100, a novel CXCR4 antagonist and hematopoietic cell mobilizer. We also demonstrate the functional interactions between CXCR4 and β_2_AR, which support the addition of propranolol to GPC-100 as a novel strategy for HSC mobilization.

GPC-100 demonstrated efficacy in disrupting CXCL12-mediated calcium signaling, cell migration and β-arrestin recruitment to CXCR4 that was comparable with AMD3100. However, GPC-100 was a better mobilizer in vivo. The competitive binding assay revealed that GPC-100 possessed superior binding to CXCR4 compared to AMD3100. We speculate that this could be due to the chemical scaffold of GPC-100 forming enhanced interactions in the CXCR4 binding pocket stabilizing the receptor differently from AMD3100. The functional consequences of ligand binding, whether activating or inhibiting, are dependent on the structural characteristics of the ligand-receptor complex [67, 68]. This influences the signal transducers such as G-proteins or arrestin-recruiting GPCR kinases, which engage in the intracellular cavity of a GPCR. For example, Jorgensen et al showed that another small molecule CXCR4 antagonist AMD11070 had a higher binding affinity to CXCR4 and greater potency to block CXCL12 induced migration than AMD3100, but AMD3100 was a better mobilizer. This was thought to be due to the biased signaling of β-arrestin recruitment by AMD3100, and differences in the binding modes [68]. Another possibility of the observed differences in AMD3100 and GPC-100 could be the assay set-up and timescale differences. For example, the binding assay captured a ‘clean’ binding event in a HEK293 cell system overexpressing CXCR4 at 1–3 hours. On the other hand, migration was measured at 4 to 24 hours and calcium flux was instantaneous in native cells with endogenous expression of CXCR4, which can lead to structural re-orientation. Additional studies are required to probe more into mechanisms leading to greater efficacy of GPC-100 observed in our in vivo studies.

GPCRs are targeted by 30%-40% of current drugs making them one of the most tractable drug targets [69]. However, only a small number (∼110 out of ~850) of GPCRs has been successfully targeted due to limited understanding of the distinct pharmacology of GPCRs that involves receptor heteromerization [70]. CXCR4 and β_2_AR are known to form heteromeric complexes with other GPCRs [42, 71–74]. The receptor heteromerization may lead to changes in ligand binding affinity, G protein-coupling, efficacy, receptor trafficking or β-arrestin interactions [75]. Successful investigation of GPCR heteromerization in endogenous, unmodified native cells has been challenging due to factors such as low protein abundance and shortage of detection methods [59]. Using a highly sensitive and specific assay like PLA, our study is the first that uses cancer cells endogenously expressing CXCR4 and β_2_AR to demonstrate that the two receptors may form heteromers or are at least in close enough proximity that allows receptor crosstalk [76]. Co-activation of CXCR4 and β_2_AR led to enhanced calcium flux and increased recruitment of β-arrestin to CXCR4. This indicates that the proximity of the two receptors has downstream functional consequences mediated by both G-protein dependent and independent pathways highlighting the importance of GPCR heteromers as druggable targets. It is known CXCR4-mediated calcium signaling regulates cell migration and survival [77]. Recent studies suggest that β_2_AR can also utilize non-canonical, G-protein independent mechanisms for inducing calcium flux [78]. Therefore, it is possible that epinephrine-induced increase in β-arrestin recruitment to CXCR4 as observed in our assay leads to a mega-complex [79] that links CXCR4-β_2_AR-β-arrestin to a calcium activating machinery. While the role of calcium flux in HSC biology is still emerging [80], calcium flux has been shown to activate the calcineurin/NFAT (nuclear factor of activated T cells) pathway and downstream gene reprogramming, leading to changes in HSC maintenance and differentiation [81]. Therefore, increase in calcium flux due to CXCR4-β_2_AR co-activation may be involved in HSC mobilization. Collectively, our findings suggest the possibility of signaling crosstalk between CXCR4 and β_2_AR, opening up the opportunity for novel therapeutic approaches.

HSC mobilization is associated with a concomitant increase in circulating WBC [82–85]. In mice, propranolol significantly enhanced GPC-100-induced WBC mobilization. This could be explained by the independent effect of propranolol on HSCs or signaling consequences from the interactions between β_2_AR and CXCR4. Previously, β_2_AR and CXCR4 were shown to interact physically and functionally leading to enhanced lymphocyte retention in the lymph nodes [27]. Similar interactions may also play a role in HSC retention by CXCR4 in BM given that both receptors are expressed on HSCs. However, due to technological limitations, such physical interactions have yet to be demonstrated in HSCs. Knight et al. and several others have discussed that propranolol can promote HSC growth and differentiation, as well as improve BM cellularity [39, 86–88]. Particularly, propranolol treatment led to upregulation of CD34^+^ HSC like gene signature and shifted cell differentiation away from myeloid bias in MM patients, which can lead to better engraftment in ASCT. Therefore, it is possible that propranolol enhanced GPC-100 mobilization by increasing the HSC yield.

Our studies show that propranolol improves GPC-100 induced HSC mobilization by 2-fold. Given that clinically GPC-100 can mobilize HSCs to PB up to 12-fold [24], the increase from the addition of propranolol to GPC-100 could provide sufficient HSCs harvest. This suggests the possibility of HSC mobilization without the use of G-CSF. It is an important finding since the elimination of G-CSF from the treatment may reduce the risk of moderate to severe side effects of G-CSF such as severe bone pain and rarely, splenic rupture [17]. MM patients have a high symptom burden and poor health-related quality of life [89] as well as history of treatments that negatively affect G-CSF mobilization. An alternative approach of using an oral beta blocker like propranolol eliminates the risks and burden created by daily subcutaneous G-CSF injections, as well as toxicity from repeated mobilization attempts. It is worthwhile to note that GPC-100 and propranolol may especially benefit patients with conditions like sickle cell disease, where G-CSF is contraindicated for stem cell collection [17]. Together, these studies suggest the advantage of adding propranolol in the HSC mobilization regimen.

Addition of propranolol to G-CSF and GPC-100 (triple combination) resulted in a significant increase in viable, functional HSCs compared to G-CSF plus AMD3100. These cells were functionally capable of differentiating into myeloid and erythroid multipotent progenitors and also expressed primitive markers known for long-term repopulating capacity of HSCs. CXCR4 and β_2_AR are expressed on most immune cells [29]. We observed that propranolol significantly increased GPC-100-induced lymphocyte mobilization. Therefore, we are currently investigating mobilization of certain immune cell subsets for potential utility in therapeutic areas such as adoptive cell therapies. Additionally, the present study was performed in naïve mice and the effects of propranolol will likely be amplified in a model wherein the BM microenvironment and HSC differentiation is compromised [90]. Future studies to determine the impact of our dosing regimen in mouse models of chronic stress or cancers like MM are warranted.

In summary, our preclinical findings propose the therapeutic co-targeting of CXCR4 and β_2_AR pathways and support the addition of propranolol to GPC-100 for HSC mobilization for ASCT in MM patients. The triple combination of GPC-100, propranolol, and G-CSF can potentially be best-in-class and target patient populations where other mobilization regimens have failed. Propranolol could prove to be a safe, accessible, and inexpensive option to supplement the mobilization therapies for greater stem cell yields in fewer apheresis sessions and reduce the financial burden on patients and healthcare systems. This is currently being tested in our two-arm Phase II clinical trial (NCT05561751) [91] with a GPC-100 plus propranolol arm and GPC-100, propranolol, and G-CSF arm.

## Supporting information

S1 File Supporting information on materials and methods. (PDF)

S2 File Supporting figures. (PDF)
