# Supplementary material for: GPC-100, a novel CXCR4 antagonist, improves in vivo hematopoietic cell mobilization when combined with propranolol
Source: PLoS One. 2023 Oct 25;18(10):e0287863. doi: 10.1371/journal.pone.0287863 (PMC10599528; doi:10.1371/journal.pone.0287863)
Supplement: S1 File — (PDF) [file pone.0287863.s001.pdf]

S1 File: Supporting information on materials and methods

GPC-100, a novel CXCR4 antagonist, improves in vivo hematopoietic cell mobilization when combined with propranolol.

S1 Method: RT-qPCR for CXCR4 and ADRB2

Total RNA was isolated using RNeasy Mini Kit (Qiagen, Valencia, CA). First-strand cDNA synthesis and RT-qPCR was performed using High-capacity cDNA reverse transcription kit and Power SYBR Green PCR Master Mix, respectively (Applied Biosystems, Foster City, CA). The primer sequences are as follows: CXCR4: 5'-CCACCATCTACTCCATCATCTTC-3' and 5'-ACTTGTCCGTCATGCTTCTC-3', ADRB2: 5'-CTCTTCCATCGTGTCCTTCTAC-3' and 5'-AATCTTCTGGAGCTGCCTTT-3', and ACTB (β-actin): 5'-GGACTTCGAGCAAGAGATGG-3' and 5'-AGCACTGTGTTGGCGTACAG-3'. The following PCR condition was used: 50 °C for 2 min and then 95 °C for 10 min, followed by 40 cycles of 95 °C for 15 s and 60 °C for 1 min, followed by 95 °C for 15 s, 60 °C for 1 min, and 95 °C for 1 s for melt curve analysis using QuantStudio 3 instrument (Applied Biosystems). The expression of CXCR4 and ADRB2 mRNAs was normalized to the expression of ACTB mRNA.

Supplemental Table 1. Expression of CXCR4 and ADRB2 mRNA measured by RT-qPCR.

Cell line	dCT	
	CXCR4	ADRB2
Namalwa	3.866	13.196
MDA-MB-231	7.69	8.09

MDA-MB-231-M1	5.78	7.28
U937	5.07	9.79
MM.1S	7.61	8

Supplemental Table 2: Reagents

Material	Catalog no.	Supplier
TagLite® Chemokine CXCR4 terbium-labeled cells	C1TT1CXCR4	CisBio (Codolet, France)
Chemokine CXCR4 receptor red agonist CXCL12 fluorescent ligand	L0012RED	CisBio (Codolet, France)
Tag-lite Buffer	LABMED	CisBio (Codolet, France)
HTRF low-volume white microplates	66PL96005	CisBio (Codolet, France)
PrestoBlue Cell Viability Reagent	A13261	Invitrogen (Waltham, MA, USA)
Rabbit anti-CXCR4 antibody (clone UMB2)	ab124824	Abcam (Waltham, MA, USA)
rabbit IgG isotype control	ab172730	Abcam (Waltham, MA, USA)
mouse anti-human β_2AR antibody (clone E-3)	sc-271322	Santa Cruz (Santa Cruz, CA)
mouse IgG2b isotype control	ab91366	Abcam (Waltham, MA, USA)
Plerixafor octahydrochloride	HY-50912	MedChemExpress (Monmouth Junction, NJ)
Propranolol hydrochloride	HY-B0573S	MedChemExpress (Monmouth Junction, NJ)
Recombinant murine G-CSF	250-05	Peprotech (Cranbury, NJ)
Recombinant human SDF-1α	300-28A	Peprotech (Cranbury, NJ)
Ulocuplumab	TAB-H72	Creative Biolabs (Shirley, NY)
Human IgG4 kappa isotype control	C0045	Crown Bioscience (Beijing, China)
Alexa Fluor 568-conjugated goat anti-human IgG	A-21090	Invitrogen (USA)

PE Rat Anti-Human CD184 Clone 1D9	551510	BD PharMingen (San Diego, CA)
PE Rat IgG2a, κ Isotype Control Clone R35-95	554689	BD PharMingen (San Diego, CA)
Alexa Fluor 488-conjugated anti-β_2AR antibody (R11E1)	sc-81577	Santa Cruz (Santa Cruz, CA)
Alexa Fluor 488-conjugated normal mouse IgG1	sc-3890	Santa Cruz (Santa Cruz, CA)
GPC-100	Custom synthesis	SciAnda Pharmaceuticals, Ltd (Shanghai, China)

Supplemental Table 3: Antibodies for LSK cells using flow cytometry

Target	Clone	Fluor	Supplier
Mouse Lineage Antibody Cocktail (CD3, Gr-1, CD11b, CD45R,Ter-119)	145-2C11. RB6-8C5, M1/70, RA3-6B2, TER-119	FITC	Biolegend 78022
CD117 (c-Kit)	2B8	PE	Biolegend 105808
Sca-1	D7	Alexa Fluor 647	Biolegend 108118
CD34	SA376A4	Brilliant Violet 421	Biolegend 152208
CD150 (SLAM)	TC15-12F12.2	BV711	Biolegend 135312
Viability	N/A	eFluor 780	Invitrogen 65-0865-18
