# Supplementary material for: GPC-100, a novel CXCR4 antagonist, improves in vivo hematopoietic cell mobilization when combined with propranolol
Source: PLoS One. 2023 Oct 25;18(10):e0287863. doi: 10.1371/journal.pone.0287863 (PMC10599528; doi:10.1371/journal.pone.0287863)
Supplement: S2 File — (PDF) [file pone.0287863.s002.pdf]

<u>**S2 File: Supporting Figures**</u>

GPC-100, a novel CXCR4 antagonist, improves in vivo hematopoietic cell mobilization when combined with propranolol

Supplemental Figures

S1 Fig

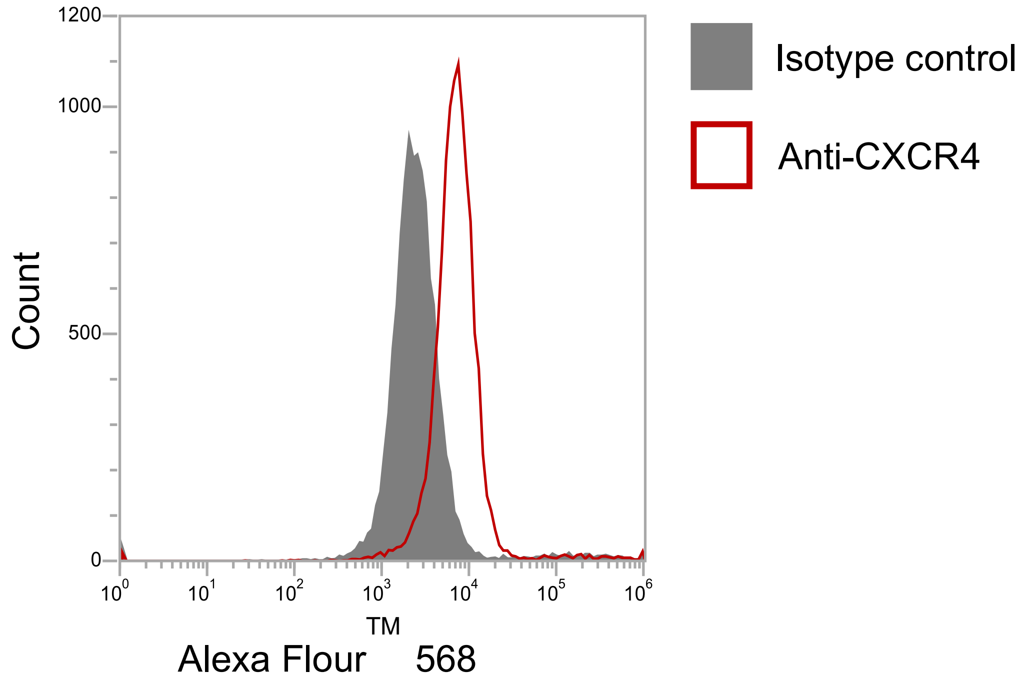

Supplementary Figure 1. Expression of CXCR4 in U937 cells. U937 cells were pretreated with Fc receptor binding inhibitor polyclonal antibody and surface expression of CXCR4 was detected by flow cytometry with isotype control or anti-CXCR4 antibody (Ulocuplumab) followed by Alexa Fluor 568-conjugated goat anti-human IgG antibody.

S2 Fig

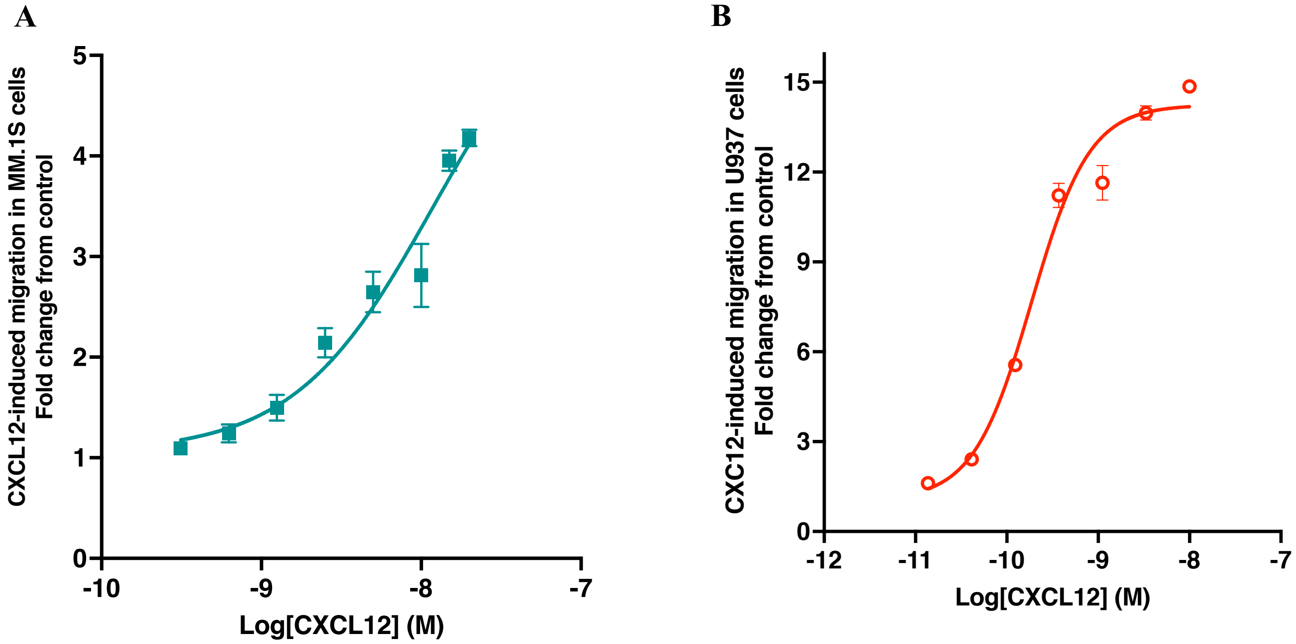

Supplementary Figure 2: CXCL-12 induced migration. MM.1S (A) or U937 (B) cells were suspended in serum-free media containing 0.5% BSA at 5X106 cells/ml and 100 µl of cell suspension was added to transwell insert. SDF-1/CXCL12 at the indicated concentrations in 800 µl of serum-free media containing 0.5% BSA was added to the lower chamber of the transwell. The transwell insert with 100 µl cell suspension was put on the lower chamber very carefully avoiding bubbles. The transwell plates were incubated at 37 °C for 4 hours (MM.1S) or 24 hours (U937) to allow cell migration.

S3 Fig

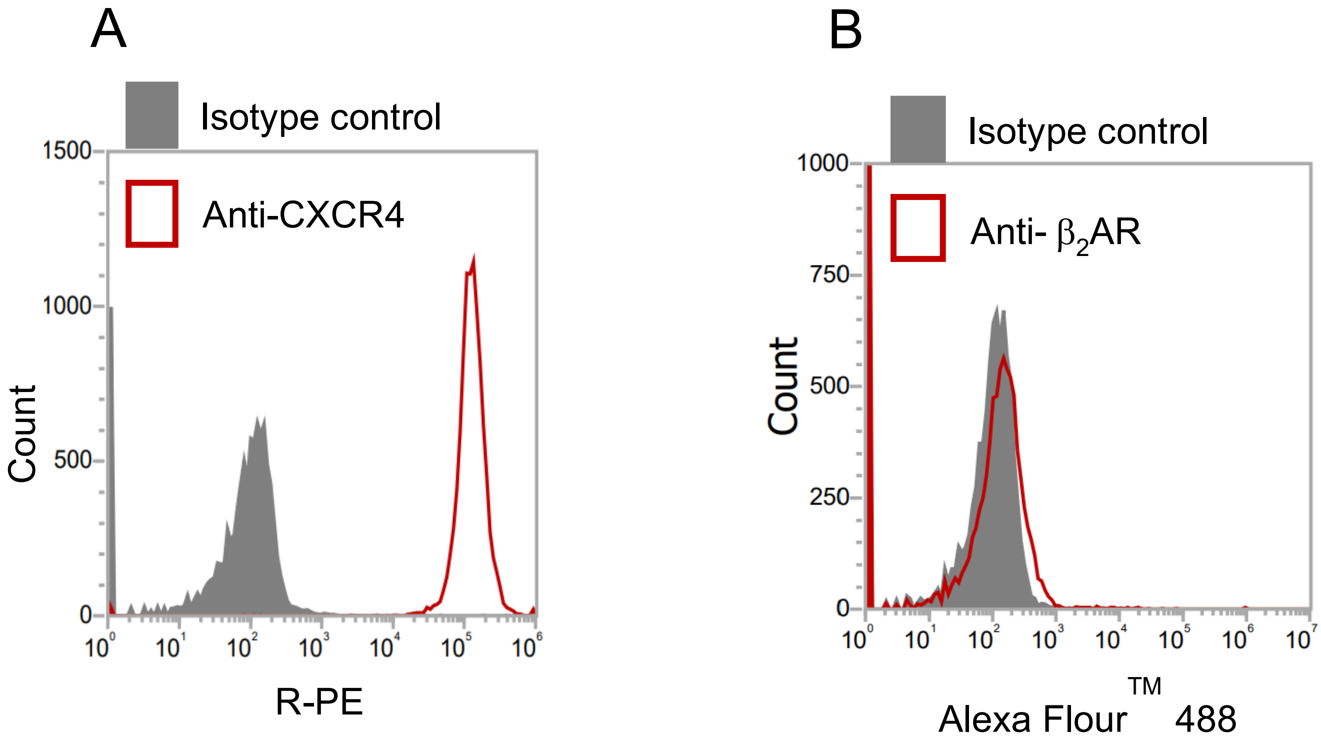

Supplementary Figure 3. Expression of CXCR4 and β_2AR in Namalwa cells. Flow cytometry was used to detect the surface expression of endogenous CXCR4 and β_2AR in Namalwa cells after staining with PE-conjugated anti-human CXCR4 (1D9) (A) and Alexa-Fluor 488-conjugated anti-human β_2AR (R11E1) antibodies (B).

S4 Fig

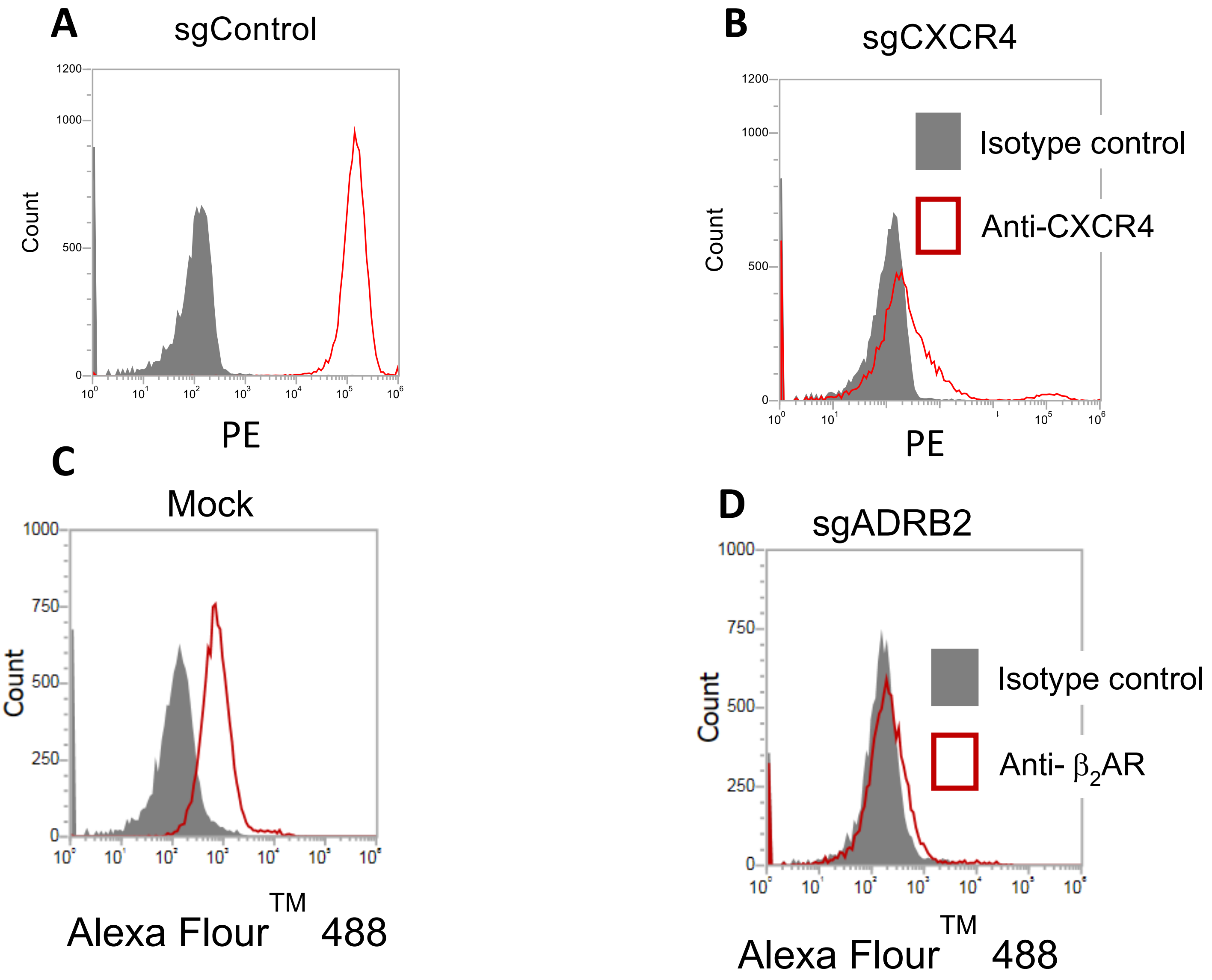

Supplementary Figure 4: Validation of Namalwa-CXCR4 knockout and MDA-MB-231-ADRB2 knockout cells. Cell surface expression of CXCR4 in Namalwa cells treated with CRISPR/Cas9-sgControl (A) and CRISPR/Cas9-sgCXCR4 was detected by flow cytometry with isotype control or PE-conjugated anti-human CXCR4 (1D9) antibodies (B). Cell surface

expression of β2AR in parental (C) and CRISPR/Cas9-sgADRB2-treated MDA-MB-231 cells (D) was detected using flow cytometry with isotype control or Alexa-Fluor 488-conjugated anti-human β2AR (R11E1) antibodies.

S5 Fig

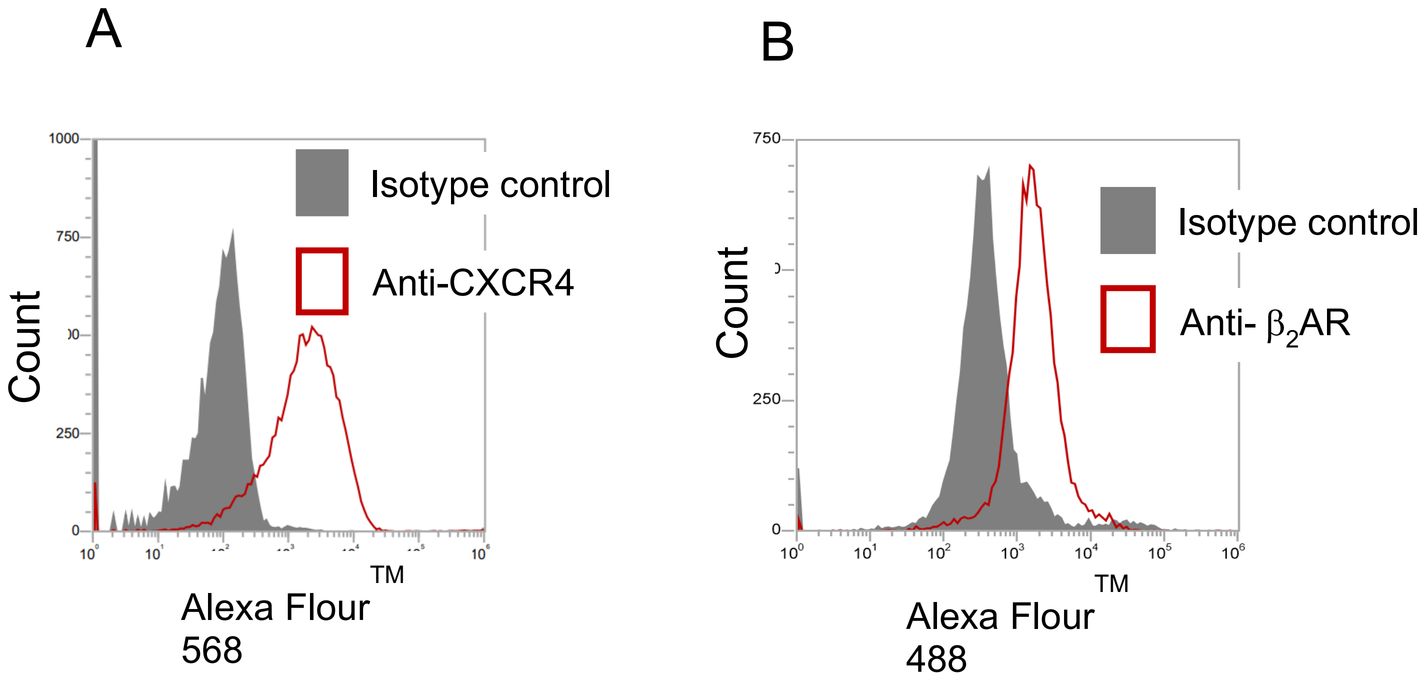

Supplementary Figure 5. Expression of CXCR4 and β2AR in MDA-MB-231 cells. Flow cytometry was used to detect the surface expression of endogenous CXCR4 and β2AR in MDA-MB-231 cells after staining with anti-human CXCR4 (Ulocuplumab) followed by Alexa Fluor 568-conjugated goat anti-human IgG antibody (A) and Alexa-Fluor 488-conjugated anti-human β2AR antibody (R11E1) (B).

S6 Fig

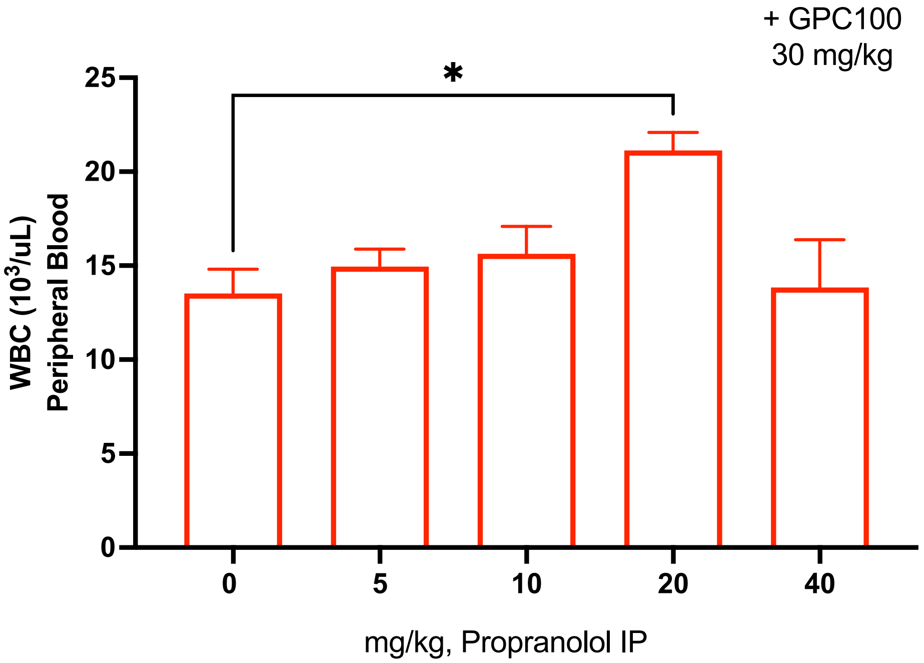

Supplementary Figure 6: Propranolol dose response for GPC-100 mobilization. Propranolol (0 to 40 mg/kg, IP) was administered for 7 days, and GPC-100 was co-administered on day 7. Data expressed as mean ± SEM. Statistical significance *p<0.05, **p<0.01

S7 Fig

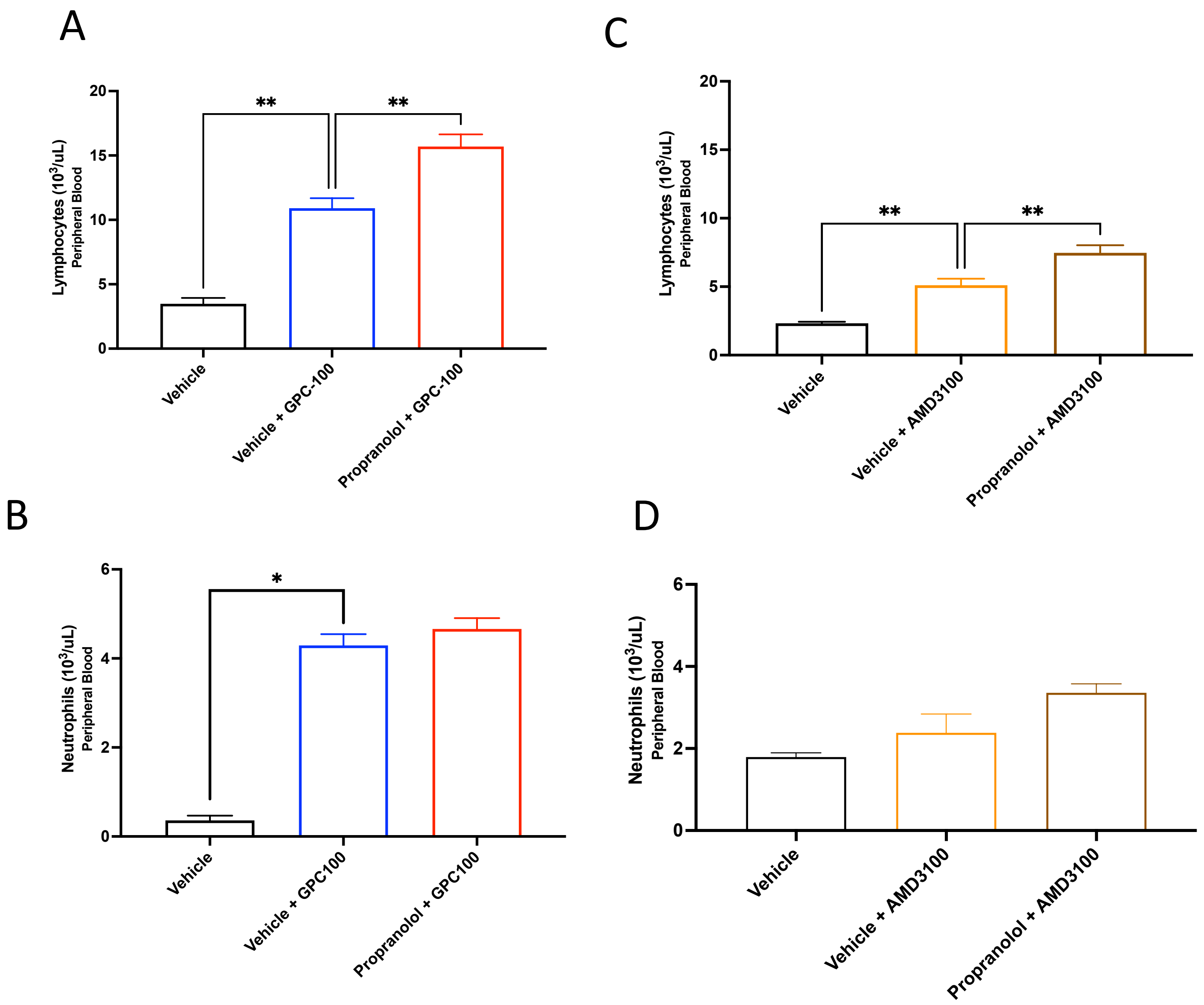

Supplementary Figure 7: Lymphocyte and neutrophil mobilization by GPC-100 and AMD3100 alone and in combination with propranolol. Propranolol (20 mg/kg, IP) was administered for 7 days and GPC-100 (A-B) or AMD3100 (C-D) were co-administered on day 7. Data expressed as mean ± SEM. Statistical significance *p<0.05, **p<0.01

S8 Fig

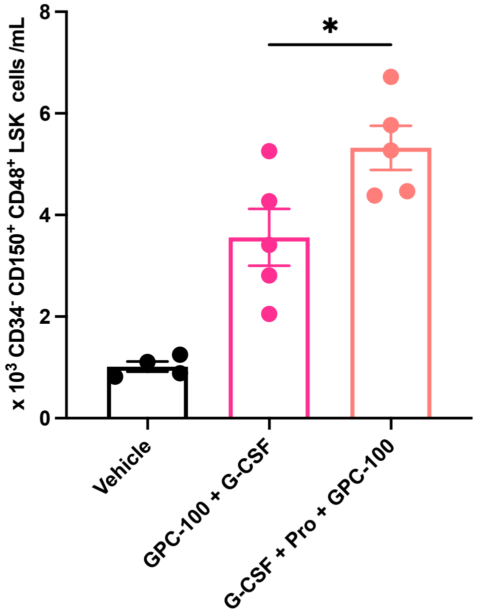

Supplementary Figure 8: In vivo LT-LSK cell mobilization. Mice were treated with propranolol (20 mg/kg, IP) or PBS (IP) for 7 days, G-CSF (0.1 mg/kg, SC) twice daily from days 2-6 and GPC-100 (30 mg/kg, IV) co-administered with propranolol on day 7. Blood was collected 2 hours later. Sca-1^+ and $cKit^+$ cells were gated from lineage negative cells (LSK cells). $CD150^+$ and $CD48^+$ cells were then selected with LSK $CD34^-$ as the parent gate. The long-tern HSC population was defined as $CD34^-CD150^+CD48^+$LSK cells.
